# Device-associated Central Nervous System Infection Caused by Candida parapsilosis

**DOI:** 10.7759/cureus.3140

**Published:** 2018-08-14

**Authors:** Gurpreet S Bhalla, Muqtadir Malik, Manbeer S Sarao, Kuntal Bandyopadhyay, Pratiksha Singh, Satish Tadepalli, Lavan Singh

**Affiliations:** 1 Microbiology, Army Hospital/Research and Referral, New Delhi, IND; 2 Microbiology, Army Hospital/Research and Referral, Delhi, IND; 3 Internal Medicine, UPMC Pinnacle, Harrisburg, USA; 4 Station Health Organization, Military Hospital, Amritsar, Amritsar, IND; 5 Internal Medicine, Hackensack Meridian - Ocean Medical Center, Edison, USA

**Keywords:** device-associated, cns infection, candida, hydrocephalus, evd, ommaya

## Abstract

Meningitis is a common and life-threatening infection of the central nervous system (CNS) in infants with long-term and disabling sequelae like hydrocephalus. Hydrocephalus is treated by diverting cerebrospinal fluid (CSF) either to another body cavity (via CSF shunt) or externally (via CSF drain) which are prone to infection. Though rare, *Candida parapsilosis* (*C. parapsilosis*) is a known pathogen in device-associated CNS infections and has been reported in both, infants and adults.

A six-month-old male infant was brought to the hospital with disproportionate head enlargement of three months duration. Magnetic resonance imaging (MRI) was suggestive of gross asymmetrical hydrocephalus. An external ventricular drain (EVD) was placed, and vancomycin and meropenem were started. Four weeks later, he developed a fever with a blocked EVD. Repeat MRI revealed gross asymmetric dilatation of left lateral ventricle along with pneumocephalus in the right periventricular region. A right temporoparietal craniotomy with drainage of a multiloculated abscess was done along with the removal of right EVD and placement of left EVD. CSF showed pan-susceptible *C. parapsilosis* and fluconazole was started. Despite treatment, CSF continued to grow *C. parapsilosis* through day 10. The EVD was removed, and an Ommaya reservoir along with the ventricular catheter was placed for better interventricular antibiotic administration. After day 13 CSF became sterile. Ommaya reservoir was removed, fluconazole was continued for three weeks, and a ventriculoperitoneal shunt was placed five weeks later.

The device-associated CNS infections are insidious with nonspecific manifestations making diagnosis difficult. *C. parapsilosis* has been increasing in prevalence, especially in immunocompromised hosts, infants, and in patients with indwelling catheters. Amphotericin B or fluconazole is the usual treatment with excellent outcomes and no mortality. This case underscores the need for suspicion of *C. parapsilosis* as a cause of device-associated CNS infections.

## Introduction

Meningitis of bacterial or nonbacterial origin is a common and life-threatening infection of the central nervous system (CNS) in infants. With the advent of modern medicine, the mortality rate has decreased with a proportionate increase in the risk for long-term and disabling sequelae. Meningitis in infants can lead to various postinfectious sequelae among which hydrocephalus is common. It can result from blockage of the cerebrospinal fluid (CSF) flow at the aqueduct of Sylvius or the outlets of the fourth ventricle, obstruction of flow within the subarachnoid spaces, or an impediment to CSF absorption.

Hydrocephalus requires hospital admission and is treated by diverting CSF either to another body cavity (via CSF shunt) or externally (via CSF drain). Both CSF shunts and CSF drains are prone to infection with higher infection rates noted in those undergoing successive shunt revisions. Gram-positive cocci account for a majority of these cases, but Gram-negative and positive bacilli, fungi, and antimicrobial resistant bacteria have also been reported [1-2]. Fungi, especially Candida species (Candida spp.), have emerged as an important pathogen in such infections as evidenced by increasing literature. Though rare, *Candida parapsilosis* (*C. parapsilosis*) is a known pathogen in device-associated CNS infections and has been reported not only in infants but also in adults [3].

 A case of device-associated CSF infection by *C. parapsilosis* in an infant with hydrocephalus is being reported.

## Case presentation

**Figure 1:**
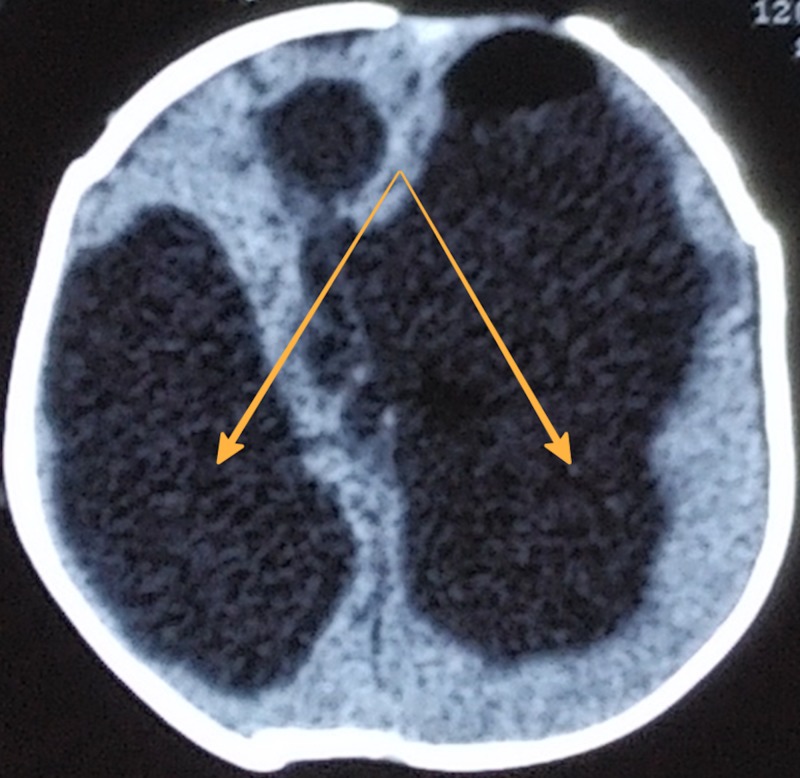
Magnetic resonance imaging scan showing gross asymmetrical hydrocephalus.

A six-month-old male infant, with a significant past medical history of neonatal meningitis on the second day of life, was brought with complaints of disproportionate head enlargement for three months duration. Initial magnetic resonance imaging (MRI) was suggestive of gross asymmetrical hydrocephalus with obstruction at the level of the aqueduct, and no signs of ependymal thickening (Figure 1).

Ventricular tap was done, and CSF was received for cytology, biochemical analysis, and culture. Cultures were sterile, and there were no features of infection.

For intra-cranial pressure reduction, an external ventricular drain (EVD) was placed, and intraventricular vancomycin (10 mg 12 hourly) was started along with parenteral vancomycin (120 mg 8 hourly) and meropenem (240 mg 8 hourly). Serial CSF monitoring was continued.

Four weeks later, the child developed a fever. It was noted that the EVD had blocked and a repeat MRI scan revealed gross asymmetric dilatation of left lateral ventricle along with air-fluid level in right periventricular region suggestive of pneumocephalus. The child was managed by right temporoparietal craniotomy and excision of multiloculated abscess done along with the removal of right EVD and placement of left EVD.

**Figure 2:**
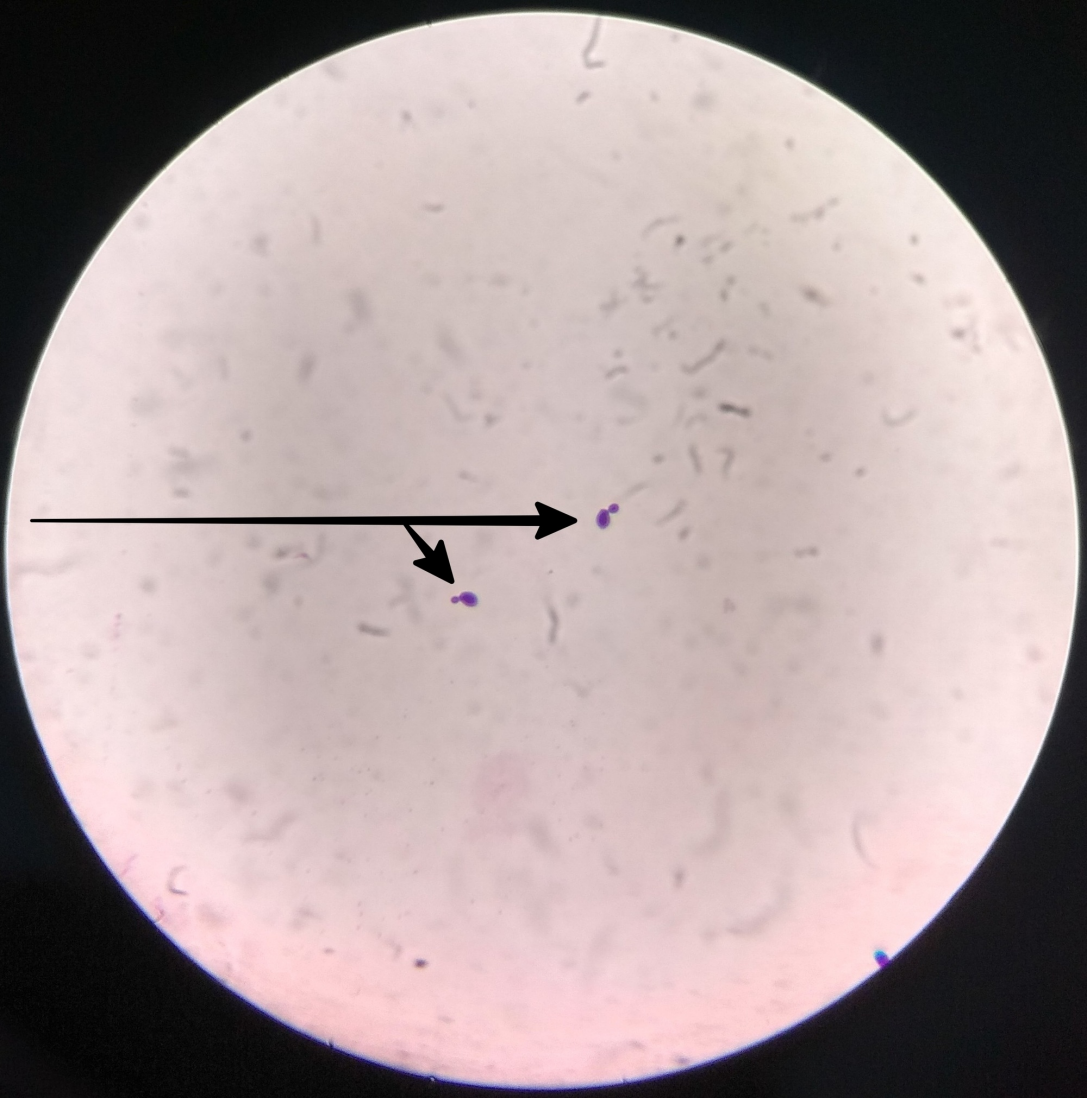
Gram stain of cerebrospinal fluid showing gram-positive budding yeast.

The CSF samples received showed features of infection and Gram-positive budding yeast was seen on a direct stain (Figure 2).

*Candida parapsilosis* was isolated from culture and was susceptible to all antifungals. Fluconazole (50 mg 24 hourly) was started, and serial monitoring of CSF continued. Despite treatment, daily CSF samples continued to grow *C. parapsilosis* through day 10. The EVD was removed, and an Ommaya reservoir along with a ventricular catheter was placed for better intraventricular antibiotic administration (vancomycin 10 mg 12 hourly). CSF samples taken 13 days and onwards were sterile. Clinically, the infant became afebrile and stable.

Antifungal therapy was continued for three weeks. The Ommaya reservoir was removed, and a ventriculoperitoneal shunt was placed five weeks later. Three months later, the infant remains asymptomatic.

## Discussion

The device-associated CNS infections have nonspecific manifestations and have an insidious nature, which makes diagnosis difficult. Retrograde infection is the most likely mechanism of infection of CSF drains. Microorganisms may enter the device by tracking from the exit site alongside the device, gaining access to the fluid column that drains CSF. CSF shunts and CSF drains are prone to infection with a reported incidence rate varying from 4% to 17% [4].

Bacteria remain the most prevalent cause of device-associated CNS infections. Though fungi are rare causes, the growing evidence suggests that fungal infections should be a differential in device-associated infections. Past studies have shown a varying incidence of shunt infections caused by fungi. Chiou et al. [5] in a retrospective study performed in 1994 reported that fungi were responsible for 17% (8/48) of shunt infections. Baradkar et al. [6] reported that 25% of shunt infections were due to fungi. Much higher infection rates with Candida spp. of 74% were reported by Fernandez et al. [7].

As in the present case, literature [8-9] mentions that 77% of Candida infections developed within three months of shunt manipulation, suggesting inoculation of the organism during the procedure. Risk factors reported for candidal device infections include the administration of broad-spectrum antibiotics, prior meningitis, CSF leakage, abdominal surgery, immune suppression and after medical device insertion. Clinical presentation of device-associated infection depends upon its location. Transient candidemia with the secondary colonization of shunts and drains have been suggested by other reports as a possible source of infecting Candida organisms [10].

As fungal infections are rare causes of device-associated CNS infections, fungi are initially not considered as the implicating pathogen. The only definitive diagnostic test is the direct observation and culture of the CSF. *C. parapsilosis* has been increasing in prevalence, especially in immunocompromised hosts, neonates, and in patients with indwelling catheters [11].

As reported by earlier studies, *C. albicans* remains the most important pathogen, followed by *C. parapsilosis* and *C. glabrata* with symptoms appearing as early as one week to as delayed as one year [5-6]. Amphotericin B or fluconazole is the usual treatment with excellent outcomes and no mortality [3, 5-7, 9].

*Candida parapsilosis* is the most common fungus isolated from human hands, and given its ability to transfer horizontally, it can contaminate medical devices with ease. A study reported hand colonization of more than 25% of healthcare workers in a community hospital with *C. parapsilosis* [12]. Thus, in patients with CNS devices, adherence to a checklist consisting of hand hygiene and appropriate skin preparation before insertion, use of sterile barriers (sterile gloves, sterile gown, cap, mask, and large sterile drape), and adherence to the policy for EVD maintenance can significantly reduce the infection rates. The use of “practice bundles” may also be valuable in the development of standardized protocols which are effective at lowering CSF shunt infection rates.

This case underscores the need for suspicion of *C. parapsilosis* as a cause of device-associated CNS infections.

## Conclusions

Device-associated CNS infections are insidious with nonspecific manifestations making diagnosis difficult. *C. parapsilosis *has been increasing in prevalence, especially in immunocompromised hosts, neonates, and in patients with indwelling catheters. It has a high affinity for parenteral nutrition, frequently colonizes the hands of healthcare workers, and forms a biofilm on prosthetic surfaces and central venous catheters. Extraventricular drainage, therapy with amphotericin B or fluconazole (intravenous or intraventricular), and insertion of a new shunt remain the principal components of the treatment regimen for pediatric fungal shunt infections. There is no established recommendation for the duration of treatment of pediatric fungal shunt infection or the role of other newer antifungal drugs. This case underscores the need for suspicion of *C. parapsilosis* as a cause of device-associated CNS infection.
